# Stepping up to meet the challenge of freezing of gait in Parkinson’s disease

**DOI:** 10.1186/s40035-022-00298-x

**Published:** 2022-05-01

**Authors:** Simon Lewis, Stewart Factor, Nir Giladi, Alice Nieuwboer, John Nutt, Mark Hallett

**Affiliations:** 1 ForeFront Parkinson’s Disease Research Clinic, Brain and Mind Centre, School of Medical Sciences, University of Sydney, Sydney, NSW Australia; 2 Jean and Paul Amos Parkinson’s Disease and Movement Disorders Program, Emory University School of Medicine, Atlanta, GA USA; 3 Movement Disorders Unit, Department of Neurology, Tel-Aviv Sourasky Medical Center, Sackler School of Medicine and Sagol School of Neuroscience, Tel Aviv University, Tel Aviv, Israel; 4 Department of Rehabilitation Sciences, KU Leuven, Leuven, Belgium; 5 Movement Disorder Section, Department of Neurology, Oregon Health & Science University, Portland, OR USA; 6 Human Motor Control Section, National Institute of Neurological Disorders and Stroke, National Institutes of Health, Bethesda, MD USA

**Keywords:** Freezing of gait, Computational modeling, Standardized definitions and assessments, Novel paradigms, Phenomenology, Pathophysiology, Treatment

## Abstract

There has been a growing appreciation for freezing of gait as a disabling symptom that causes a significant burden in Parkinson’s disease. Previous research has highlighted some of the key components that underlie the phenomenon, but these reductionist approaches have yet to lead to a paradigm shift resulting in the development of novel treatment strategies. Addressing this issue will require greater integration of multi-modal data with complex computational modeling, but there are a number of critical aspects that need to be considered before embarking on such an approach. This paper highlights where the field needs to address current gaps and shortcomings including the standardization of definitions and measurement, phenomenology and pathophysiology, as well as considering what available data exist and how future studies should be constructed to achieve the greatest potential to better understand and treat this devastating symptom.

## Introduction

Freezing of gait (FOG) impacts most patients with advanced Parkinson’s disease (PD) [1] and despite significant efforts, our current treatments are often unable to prevent sufferers from losing their independence. Indeed, it could be argued that the observations derived from previous reductionist research techniques focusing on understanding components of the phenomenon (e.g., imaging, neurophysiology, and epidemiological observations) are precisely the reason that we are failing to achieve a good understanding of how FOG arises in the first place. Therefore, novel approaches are required if we are to achieve real progress that would result in better treatments. Rather than focusing on limited ‘correlational’ data, the field needs to integrate large amounts of data that are collected concurrently. Indeed, a Scientific Issues Committee convened by the International Parkinson’s and Movement Disorders Society has recently proposed the pursuit of a Systems Biology approach [2, 3]. Whilst it is clear that in PD, the use of techniques such as computational modelling is only in their infancy, work exploring a range of issues including the influence of dopamine on basal ganglia function, the origin of beta-band oscillations and the therapeutic actions of deep brain stimulation (DBS) has already begun (for review, see Humphries et al. 2019 [4]). Clinicians will need to work closely with colleagues from different backgrounds including engineering and information technology, so that this data can undergo complex computational processing to produce quantitative models that can then be tested back in the clinic through an iterative process. Thus, it is vital that experts working in the field generate appropriate data that can inform the model without oversimplifying the problem. These efforts will need the constant bi-directional flow of information between experimentalists and theorists to allow for refinement and reality checking of the emergent properties that arise from the models being proposed. This paper highlights the key components of our existing knowledge and identifies how these seemingly disparate pieces of information could be better studied and integrated in a novel comprehensive framework to achieve better outcomes for our patients.

## Standardising definitions, assessments and measurements

One of the major challenges that needs to be addressed before any successful modeling approach could be implemented would be to standardise the definitions, assessments, and measurements used by researchers in the field. To achieve these goals, a coordinated approach that is overseen by an international consortium who can harmonise research efforts with a pre-determined common goal, is needed.

### Definition

The current definition of FOG was developed as part of a consensus paper arising from the first International Workshop organised by the National Institutes of Health in 2010 [5]. It defines FOG as “*a brief, episodic absence or marked reduction of forward progression of the feet despite the intention to walk*”. Unfortunately, there are intrinsic ambiguities with this definition, which lead to difficulties in standardised assessment, such as: what is "brief"; how should an "episode" be defined that would separate it from a continuous performance deficit; and, what qualifies as "marked reduction"? Furthermore, it is not clear if this definition should be revised to accommodate broader freezing phenomena, which can occur across body parts during a range of repetitive movements (e.g., stepping in place, upper limb movements, speech). In addition, the current definition of FOG pays little regard to any phenotypic variation; for instance, it pays little attention to the high-frequency movement phenomena that are characteristic co-manifestations that occur during most gait freezing. Indeed, of the three FOG phenotypes described in the literature to date, ‘akinetic FOG’ (displaying no discernable movement) is considered the least common type, whereas the other two phenotypes in which high-frequency events are frequently observed, namely, FOG with trembling in place and FOG with small shuffling steps interrupting more normal gait, are far more common. These different FOG phenotypes may reflect different underlying pathophysiologies but can all occur in the same individual at differing times under different circumstances, which ultimately may have differential treatment responses[6]. Furthermore, a greater focus needs to be directed to the range of FOG triggers (e.g., start hesitation, turning, dual-tasking, and doorways), as well as ‘relievers’ (e.g., cueing, climbing stairs). Such considerations are vital if we are to understand the neurobiological underpinning of these seemingly related but distinct phenomena and to arrive at better treatments.

Therefore, updating the definition of FOG represents a priority with the stated aim of distinguishing FOG from festinating gait and incorporating the broader phenotypic spectrum that ideally includes objective measures [7]. One way forward to achieve a unified definition would be to utilize a Delphi panel of experts. The panel would begin with a critique of the current definition, potentially using video recordings collected in different circumstances, specifying that an optimal definition should have direct consequences for assessment, and specific questions like those asked here. Based on answers, the definition would be tentatively revised, and there would be at least a second round of expert comment. The hope is that the definition would converge to a consensus that could be implemented globally for use in clinical practice, as well as in basic and applied research. Furthermore, consideration could then be given to producing an online resource through which patients themselves could improve their self-evaluation of FOG.

### Assessments and measurements

In parallel with addressing the need to improve the definition of FOG, there is a pressing need to develop standardised assessments across researchers to facilitate observational and interventional multi-centre studies. Measures of self-reported FOG based on questionnaires generate high test–retest measurement errors [8]. In early PD, patients hardly recognise their brief FOG episodes; while at the other end of the spectrum, interference with self-perception may occur when the symptom becomes very severe but there is also concomitant cognitive dysfunction. Therefore, such measures are not useful for observational or interventional studies and offer little that could be useful for computational modeling.

Currently, the most rigorous data that could be applied to computational modeling must be obtained from assessments conducted in the clinic, but this often fails to represent the real world experience. Whilst some ‘simple’ assessment paradigms have been validated [9, 10], the current gold-standard performance measure of FOG is percentage time frozen (%TimeFOG) during standardized FOG-provoking protocols and expert visual scoring of the ensuing episodes. This metric, derived from manual event annotation of video-vignettes, can be standardized with an off-line software, some of which have been made available via open-access platforms [11]. However, this process is time-consuming, subject to inter-tester error for actual episode delineation and based on diverse protocols [12]. One approach to addressing these limitations would be through standardized video recording assessments that could be automatically scored by deep-learning methods [13].

Even where gait testing has been designed to mimic daily life, the %Time FOG does not reflect the real-world impact of FOG. Therefore, studies are needed to first optimize the gold standard with a universal testing paradigm, incorporating an improved definition of FOG, and then to validate technology platforms for home-based measures under standardized, as well as free-living conditions against that criterion. It is possible that future studies could capitalize on ‘smart home’ environments, embedding multi-camera systems (‘living labs’) [14], which could potentially deliver a new gold-standard measure of FOG. Furthermore, the field should strive to assess these video recording methods alongside other technologies, such as marker-less motion capturing, mobile video-systems, and Wi-Fi motion detection software [15] to determine the optimal conditions to obtain the most useful data for modeling.

Due to its illusive nature in the laboratory, measuring FOG accurately in the home setting is crucial for the research agenda and would provide vital data for novel modeling approaches. Due to their wearability and general acceptance by patients, Inertial Measurement Units (IMU) could offer automated FOG detection over multiple days/weeks at home [16]. Indeed, many studies have already tested IMUs for FOG detection using a wide array of unobtrusive sensors on various body parts and many calculation methods, ranging from simple thresholds to machine learning approaches [17]. Most algorithms have been able to classify FOG-events, as well as to discriminate between groups with and without FOG with promising performance (for review see Mancini et al. 2019 [16]). Notably, neural network methodologies have achieved the most accurate classifications, with person-specific models outperforming person-independent ones [18]. However, when an automated algorithm detection was compared with the gold standard, i.e., expert-based %TimeFOG in the laboratory, good agreement was only found for long episodes of gait arrest [12]. This partially reflects the fact that the accuracy of most IMU-algorithms partly relies on frequency-based analysis of the ‘signature’ high-frequency motions of FOG, as well as the sliding time windows implemented by these systems. To reduce this problem, high agreement with clinical video-ratings has been demonstrated in recent work on insoles that can record foot pressure data with a 3D sensor collected during standardized testing in the home and in the laboratory [19]. Therefore, this patient-friendly methodology holds great promise for detecting different phenotypes of FOG in the home environment, which could provide more real-world data for computational modeling, helping to explain the commonly observed heterogeneity potentially down to the level of specific triggers/relievers in the individual.

Given that much of the strength of complex mathematical modeling lies in its ability to process large datasets from different sources, future consideration should also be given to coordinating studies that optimize recordings from multimodal systems. Some evidence for this approach can already be seen in recent work that has combined electroencephalography (EEG), electromyography sensors (EMG), and accelerometry to improve FOG detection [20]. Thought should also be given to standardizing recordings from other relevant signals, such as cognitive function and anxiety (potential of using electrocardiography—pre-print Cockx et al. 2021 https://doi.org/10.21203/rs.3.rs-735366/v1), which, if not causal contributors, are strongly correlated with FOG [21]. Thus, studies that could collect non-invasive physiological parameters, such as heart rate variability and skin conductance in conjunction with other systems (IMUs, pressure sensor, EEG, EMG) may prove highly informative when constructing multi-dimensional models, as well as when potentially considering more invasive recordings (e.g., sensing deep brain electrodes). Finally, there has been a failure to recognise that the emergence of FOG is likely to be more gradual and fluctuating with disease progression and medication intake. As such, the next generation of studies should reflect this gradient, rather than approaching FOG as a binary phenomenon, which will also provide more accurate modeling.

## Freezing of gait and balance disturbances: lumping versus splitting

The concurrence of FOG and balance disturbances in advancing PD is hard to ignore but for the purposes of computational modeling, knowing if these features are related neurobiologically or are discrete, is of critical importance. Previous studies have identified the overlap between poor balance and FOG [22], as well as identifying that a deterioration in balance may be a useful predictor for those patients developing FOG [23].

Anatomically, it would seem intuitive that the neural pathways serving gait and balance do demonstrate a degree of meaningful overlap that could link these processes but this does not necessarily represent a fixed anatomical connection and may perhaps be more functional. For example, dopamine loss is the hallmark of PD but can also be seen in some people with vascular parkinsonism and normal pressure hydrocephalus, who also experience FOG and falls [24, 25]. Indeed, whilst both FOG and balance disturbances are frequently related in PD, they can occur independently, suggesting that their pathophysiologies may, to some degree, be separable. In one recent study, PD patients reported that 61% of falls were due to FOG rather than being attributed to slips, trips, balance loss, or syncope [26].

The link between poor balance and FOG probably relates to dynamic postural control, which is defined as the ability to control the center of mass (CoM) during continuously changing conditions, including the transfer of body weight between the legs when engaged in walking. Obviously, the common FOG-triggering situations, such as turning and gait initiation, are associated with dynamic postural instability given the increased requirements to control the CoM. Such weight-shifting unloads one leg, allowing that swing leg to be lifted off the ground. When initiating gait, these movements are associated with anticipatory postural adjustments (APAs), which allow for an even more precise control of the CoM. Although not a universal finding, many studies have demonstrated that these APAs are relatively hypometric in PD patients with FOG. Indeed, when gait initiation moves the centre of mass forward beyond the limit of stability, such that a step is required to remain upright, one may stimulate the swing leg to tremble without taking a step. This alternate leg trembling has the temporal pattern and kinematics of repeated anticipatory postural responses, suggesting that the FOG associated with start hesitation is a failure to connect the APA to the stepping program [27] during an effort to move forward. However, there is a significant body of literature highlighting that APAs and stepping programs are thought to be separate processes (for review see Massion 1992 [28]). These observations suggest that research efforts focusing on gait freezing will inherently have to incorporate specific studies to identify underlying axial disturbances, especially in the context of the start hesitation sub-type of FOG.

To inform a novel computational modeling approach, several methods could be undertaken to identify and potentially isolate the role of balance in FOG. Firstly, future studies in PD patients with FOG and/or impaired balance could be conducted with and without partial weight support from ceiling track systems combined with physiological measures of anxiety (e.g., heart rate, skin conductance) to determine the respective roles of increasing balance demand mechanisms and the impact of anxiety on FOG. Secondly, patients with FOG could be instrumented with inertial sensors to determine if the fast activity of leg trembling could be recorded during situations such as walking or turning, and whether this activity alternated between the legs, as would be expected during repeated weight-shifting. Thirdly, combining large, standardised datasets from centres that have performed detailed, instrumented assessments of both FOG and balance could provide greater insights into the possible correlations between these problems. One final approach that could yield useful information would be to identify the outcomes of dedicated rehabilitation programs directed specifically at FOG and balance impairments in PD. Clearly, any responses to specific strategies that could be mapped to objectively obtain clinical, neuroimaging, and neurophysiological outcomes would provide useful information to modellers.

## Understanding phenomenology and pathophysiology

Despite an abundance of clinical experience from direct observations, there are still a significant number of fundamental gaps in our understanding of FOG despite the proposal of several pathophysiological models (for review see Giladi and Nieuwboer [29]). These range from the very basic grasp we have on phenomenology through to the more nuanced appreciation of its underlying neurobiology that manifests clinically.

### Insights from other pathologies

Whilst it is widely acknowledged that FOG is not unique to PD and is frequently observed in atypical parkinsonian syndromes, high-level gait disorders, normal pressure hydrocephalus, vascular diseases, and other neurodegenerative diseases [30, 31], it is unclear as to whether they share common pathophysiology. Furthermore, due to a lack of well conducted, large-scale observational studies, many basic elements of FOG, such as the frequency/duration of episodes, gait pattern generation, imbalance and the impact of cues are not well characterised across these other freezing conditions. Clearly, not all of these disorders have a profound loss of dopamine or response to treatment, but most are described as parkinsonian, potentially reflecting disturbances in motor networks that may be structural or functional.

These non-PD groups are clearly challenging to recruit and study. Therefore, future prospective studies with harmonised data collection across multiple centres to achieve sufficient statistical power should be constructed with open de-identified data sharing. Simple clinical data (e.g., motor, cognitive, and psychiatric assessments) collected across all subjects may highlight hitherto unrecognised relationships. A more rigorous examination, in a smaller number of patients at dedicated centres, should include detailed gait kinematics and neuromechanics with simultaneous measurements of axial motion (dynamic posturography) and EMG [32], along with standardised neurophysiological (e.g., ambulatory and seated EEG) and neuroimaging (e.g., structural/resting state MRI, dopamine/dopamine transporter imaging) data collection. Comparison of these data with the FOG and balance disturbances observed in PD would allow modellers to build more accurate network models that capitalise on real-world perturbations across clinical, neurophysiological, and neuroimaging data to help identify the relevance of contributing pathways.

### Insights from disease progression and treatment

Given the progressive nature of FOG, any complex modeling approach will require high-quality longitudinal data that record motor and non-motor features, as well as medication use. Clearly, the increased prevalence of FOG with disease duration might suggest a dopaminergic aetiology [33], but it must be appreciated that with disease progression there will also be increasing pathology across multiple neurotransmitter systems and a breakdown in the functional/structural connectivity across disseminated brain networks [34, 35]. Furthermore, it has also been suggested that increasing dopaminergic therapy in the advanced stages might have a causal role in the development of FOG, particularly in relation to the rare phenomenon of ON FOG [36].

To address the respective roles of disease progression and medication use, large collaborative studies with standardized data collection and recording are required. A variety of observational and interventional studies could be considered, each of which would have significant issues regarding feasibility. Perhaps most simply, one could envisage leveraging from planned prospective natural history studies like the Parkinson Progression Marker Initiative 2.0 (PPMI 2.0—NCT04477785), which will establish a deeply phenotyped cohort assessing the progression of clinical features, digital outcomes, as well as imaging, biologic, and genetic markers in study participants with de novo PD, prodromal PD, and healthy controls. The addition of more extensive gait assessments to a dedicated study like PPMI 2.0 with additional cognitive, affective, autonomic, sleep, and daily physical activity measures, combined with regular, standardised assessments at home and in the office as discussed above, would improve our understanding of the protective and provocative factors for developing FOG. Such a study could be conducted in newly diagnosed patients or potentially in a more enriched cohort of patients, five years from initial diagnosis who have an ‘at-risk’ phenotype (e.g. anxiety, non-tremor dominant, impaired repetitive motor task performance, executive impairments) with a higher likelihood of transition from being non-freezers [37, 38]. One further approach could be an interventional study where newly diagnosed (drug naïve) patients would be randomised to different treatment arms to explore the role of L-dopa, dopamine agonists, and monoamine oxidase-B inhibitors. This approach could be utilised to determine the differential impacts of delayed initiation of L-dopa and dopamine agonists versus those starting therapy at diagnosis, and the effect of the dosing level. Indeed, it has been hypothesised that levodopa may induce maladaptive plasticity in the striatum, which disproportionally increases the mismatch between motor and non-motor (cognitive and limbic) loops, leading to gait freezing (the levodopa paradox) [6]. Previous lines of evidence from the pre-levodopa era and observations in third-world countries would seem to refute this assertion [39]. Constructing the necessary prospective study to address this issue would prove prohibitively expensive and would obviously pose significant challenges for recruitment and retention. Therefore, retrospective chart review may offer a more pragmatic approach (see below). It would seem unlikely that even such an interventional trial would help our understanding of ON FOG, which is a rare phenomenon where there is a worsening of FOG following L-dopa [40]. Previously, ON FOG has been addressed by kinematic studies in the ON and OFF states during an appropriately rigorous levodopa challenge, including serum levodopa levels [41], but longitudinal assessments are now required to determine whether OFF FOG evolves into ON FOG (where freezing is seemingly caused by levodopa), ON–OFF FOG (where FOG persists in the ON state) or if they develop and evolve separately. These insights would have specific consequences for any modeling approach, as well as our definition of the phenomenon and its treatment [42].

### Insights from non-gait freezing

One critical aspect that could be exploited in our understanding of FOG is whether freezing is restricted to gait or represents a more universal phenomenon. The concept of ‘motor blocks’, where sudden episodes of motor breakdown are provoked by repetitive upper and lower limb tasks, as well as by speech sequences has long been recognised [43, 44]. These freezing episodes in other effectors also typically present with faulty initiation-termination responses, particularly when progressing towards the end of an automated motor sequence. When patients who experience FOG are required to perform declining movement amplitudes at fast speed within self-initiated sequencing tasks, there seems to be a consistent degradation of the neural coding of movement cycles, triggering episodes of motor output breakdown. Regardless of the functional activity, impairments in the accurate coding of the motor network appear to disable the normal motor re-initiation. For example, one recent study that required patients to perform accelerated weight-shifting sequences without stepping, demonstrated greater disturbances in freezers compared to non-freezers, which were exacerbated in OFF [45].

These behavioural observations regarding non-gait freezing have prompted efforts to identify any common neural correlates. Previously, neurophysiological studies have described beta oscillations as the ‘idling state’ of the brain and that voluntary movement requires a desynchronization of this activity. Significantly, volitional movements in PD are associated with impaired desynchronisation in these beta oscillations [46], and pathological beta activity has been identified within the subthalamic nucleus of PD patients with FOG [47]. Indeed, suppression of this beta activity either by open- [48] or closed-loop [49] DBS has been shown to ameliorate freezing episodes either during normal walking or when stepping in place. Furthermore, recent work recording subthalamic activity from chronically-implanted DBS electrodes has identified that FOG is characterised by a low-frequency cortical-subthalamic decoupling, which is lateralized to the hemisphere with less striatal dopaminergic innervation [50]. Analogous to these findings in gait, a study utilising cortical EEG to investigate a finger sequencing task has shown that compared to non-freezers, PD patients with FOG have increased beta oscillations (i.e., reduced desynchronisation) in the supplementary motor area prior to volitional movements [51]. In addition, a separate EEG study examining the effect of a dual-task on finger tapping has highlighted that increases in prefrontal beta-band synchronization are predictive of upper limb freezing [52]. Thus, as well as highlighting the neurophysiological similarities between upper limb and gait freezing, this study also underscores the potential contribution of prefrontal executive dysfunction, which has been described in FOG [53, 54]. The role of dopaminergic pathways in non-gait freezing has been less well explored to date. One study reported that dopamine replacement did not influence the frequency of events during wrist flexion/extension [55], whilst a virtual reality (VR) gait paradigm where patients utilized foot pedals has shown amelioration of freezing-related phenomena [56]. Further work utilizing more automatic finger movement or handwriting paradigms [57] is required to confirm these observations.

Despite these overlapping neurophysiological features, freezing in the upper limbs has been observed in a substantial proportion of non-gait freezers [57], suggesting that this phenomenon may capture a substrate of FOG but not the full picture. However, it should be highlighted that in a recent prospective cohort of 60 non-freezers, assessed prospectively for two years (12 convertors), repetitive finger tapping was found to identify those patients at risk of developing FOG [38] and is therefore worthy of further consideration.

Paradigms do exist to assess non-gait freezing that could specifically explore trembling in place, the sequence effect, and the role of treatment (e.g., medication and DBS). Force sensors, keyboards, and smartphone apps can all be used to quantify motor blocks during foot pedalling [58–60] and alternating hand/finger tapping [61, 62]. Indeed, many studies have been conducted utilising a VR gait paradigm where freezing episodes recorded from foot pedal movement have been correlated with actual FOG [58]. To date, combining this VR gait paradigm with fMRI has identified the neural correlates of freezing [63, 64] and related triggers including turning [65], doorways [56, 66], and dual-tasking [67]. Furthermore, this technique has been utilized to record multi-unit activity in the subthalamic nucleus (STN) during DBS surgery and demonstrated a pathological surge of beta activity prior to the onset of a freeze that differed from the recordings associated with volitional stopping [68]. In addition, this beta-band activity was unidirectionally and selectively linked with STN theta activity, which in turn was unidirectionally and selectively linked with the 3–8 Hz trembling in EMG activity seen in the lower limb muscles that activated the foot pedal. Similar to manipulating lower limb conditions for eliciting FOG, a simple test whereby subjects have to vary the size of writing strokes to fit in a funnel figure at fast speed, has also been able to elicit finger freezing episodes that can be correlated with self-reported FOG [57].

Therefore, these non-gait freezing paradigms are potentially valid models for studying FOG with multi-modal techniques. Additionally, repetitive tasks can be remotely employed via telemedicine platforms, which are relatively inexpensive and could serve as safe proxy markers or predictors of FOG, providing significant data for future modeling work. Thus, these non-gait approaches appear to be capable of providing information about the circuit mechanisms that account for the manifestations of FOG, which could be implemented by systems biologists to construct testable models.

### Insights from reductionist observations

As highlighted above, many reductionist studies comparing patients with and without FOG across clinical features and a range of biomarkers (e.g., MRI, DBS, EEG, PET) have provided insights into the role of many different physiological processes and anatomical regions. For example, a lesion analysis performed in a series of 14 patients who developed FOG, demonstrated discrete disturbances in the cerebellar locomotor region (CLR), an area functionally connected to the dorsal medial cerebellum [69]. Work from a recent meta-analysis of neuroimaging studies in PD has also suggested that CLR activation may play a compensatory role in locomotion [70]. As outlined above, other studies have suggested a more generalized pathophysiology, which has allowed a common final pathway to be postulated [71, 72]. However, the question must be raised as to whether all the relevant anatomical regions work together to produce a common input to this pathway or whether they speak separately to this single common pathway or multiple pathways.

Whilst not anatomically connected, disseminated regions of the brain are functionally connected to each other and can therefore exert influence. For example, it might be proposed that the common final pathway for FOG is associated with impaired corticothalamic and corticostriatal networks that lead to an increase in pallidal inhibitory outflow (globus pallidus internus—GPi), which is often accentuated by glutamatergic input from the STN in the presence of increased response conflict, leading to the emergence of 5–7 Hz oscillations between the two nuclei (STN-GPi). The STN activity also leads to impaired cerebellar output [71]. Ultimately, the increased pallidal outflow manifests as impaired coordination of flexor–extensor pairs in the lower limbs, leading to gait arrest. Conceptually, if there was a significant burden of pathology directly affecting this common final pathway, there would be a more pervasive gait disturbance manifesting with more constant FOG or other gait disturbances. In addition, there are many nodes that feed into this locomotor network (e.g., cortical regions dealing with conflict resolution) that may have varying degrees of input depending on circumstances. At times, these input nodes will fail and trigger the common final pathway, whereas there may be strategies to compensate for this demand such as focusing attention (e.g., cueing). To accurately model these connections, the input of observations obtained from our current reductionist datasets, such as those from neuroimaging and neurophysiology, will be needed, and then systematic perturbations, both inhibitory and facilitatory, should be applied to determine whether the prediction matches the observation. For example, to explore the neural underpinnings of cueing, patients could undertake multi-modal experiments such as simultaneous EEG and fMRI, where a VR environment has sections with and without lines presented on the floor. Such approaches would be able to probe the neural networks (imaging) and dynamic power spectra (neurophysiology) in patients with and without FOG. Behavioural data could then be fed back into computer models manipulating these neural parameters to make predictions about how cueing may ameliorate FOG.

Going forward, studies that can collect multi-modal data from the same patients to inform the modeling approaches are required, along with constant validation approaches. Patients undergoing DBS represent a unique opportunity to record from within the brain and with the advent of sensing-stimulating devices used in many centers worldwide, it will become easier to repeat longitudinal assessments over time. However, it should be noted that most patients undergoing DBS are not usually severe freezers, and rare cases of FOG following DBS have been reported [73]. In addition, MRI in such patients is challenging post-implantation and DBS signals can create significant artefact with concurrent EEG. Therefore, other prospective patient cohorts, as described above, might offer greater utility, especially if there was an opportunity to implement novel neurophysiological and neuroimaging approaches. For example, newer MRI methodologies to accurately image brainstem nuclei by detecting iron and neuromelanin content may be very useful and appear highly reproducible [74]. In addition, PET studies exploring the role of dopaminergic and cholinergic systems, as well as amyloid burden, have been published [75–78]. Other PET work has measured cerebral glucose metabolism with gait tasks performed during the uptake time of the radioligand ^18^F-fluorodeoxyglucose to explore the corticobasal-thalamocortical circuitry implicated in FOG [79]. Meanwhile, work on noradrenergic and serotonergic neurotransmitters is lacking and represents a real gap for modeling FOG. Finally, whilst ambulatory EEG has been generating useful data for modeling FOG, there probably needs to be a greater emphasis on developing other dynamic imaging techniques. Some work in FOG has been conducted using functional near-infrared spectroscopy (fNIRS) to examine changes in oxygenated-haemoglobin levels that occur in the gait assessment of patients with FOG, but obviously the region of interest is often limited to the forehead, offering little insight into the rest of the brain’s activity [80, 81]. Novel multi-optode fNIRS systems are now increasingly being used covering wider brain areas and guidelines to reduce artefacts are also available [82]. Finally, it is not clear yet whether micro-dose ambulatory PET will prove useful, but clearly, being able to measure ligand activity during gait and balance tasks may prove incredibly helpful for understanding systems biology [83].

## What other data do we have or need?

There would appear to be at least two further major obstacles to advancing the field, which could be summarised as not using data that we already have and not having a platform to test some of the ideas that need to be explored.

### What could be done with existing data?

We currently have extensive datasets that are not being utilised to their full potential (e.g., clinical, accelerometry, kinematics, MRI, PET, EEG, EMG, DBS). Establishing global collaborations between researchers in the field to work together and with bioinformaticians and bioengineers who can implement artificial intelligence and machine learning techniques may identify new discoveries within our existing data, such as risk factors/markers, and characterization of freezing episodes (e.g., onset/offset).

The variability inherent to existing biobanks, especially with regards to different scanning protocols and hardwares, can be offset to some degree by bringing together smaller well-developed datasets from researchers with well characterised cohorts to generate an open-source database, incorporating harmonized gait protocols, which are sensitive to change over time. Obviously, a standardized approach going forward using such a platform could collect prospective data, and whilst biobanks of remotely monitored, free-living gait data would be the aspiration, they would require a significant investment in the necessary infrastructure. Just as importantly, such a database will not contribute to our understanding of FOG unless ‘ground truth’ validation of its ability to capture this complex phenomenon can be ensured.

### Health data linkage

One of the recent advances in our understanding of PD generally relates to how looking at large datasets can highlight promising new avenues for disease-modifying treatment. For example, registry data have revealed that the use of beta agonist medications reduces the risk of developing PD, whereas beta blockers increase the risk, potentially acting through an effect on the alpha-synuclein gene [84]. Similarly, the use of dipeptidyl peptidase 4 inhibitors and/or glucagon-like peptide-1 receptor agonists is associated with a lower rate of PD compared to the use of other oral antidiabetic drugs [85]. Such well-constructed studies have not been undertaken to identify whether any particular medications may increase or decrease the frequency of FOG. One single-centre study with less than 200 PD patients did highlight a trend towards an increased prevalence of FOG with dopamine agonist use and a reduction in those on amantadine, but this did not survive correction for multiple variables [86]. As highlighted above, whilst there would be some obvious limitations in terms of accurately identifying FOG in community-based patients, this approach could be worth pursuing, especially where there was an a priori hypothesis. For example, the fact that anxiety has been identified as a predictor of FOG [37] may have implications for the rates of anxiolytic use amongst these patients and of course, one might anticipate the reduced use of anti-tremor agents given the motor phenotype of patients developing FOG. Finally, whilst it would be expected that increasing dopaminergic therapy would be associated with FOG, identifying any other centrally acting agent could provide novel insights into the pathophysiology (e.g. serotonergic, noradrenergic).

### Animal platforms

Despite several attempts, we lack a cogent animal model of FOG with sufficient utility to be beneficial for understanding pathophysiology and developing therapies. The non-human primate (NHP) is the only animal model that has demonstrated any FOG, which resulted from a severe dopaminergic lesion [87]. Work has been conducted in the MPTP-intoxicated NHP, which was not sufficient to induce freezing but when these monkeys underwent stereotaxic lesions in the bilateral pedunculopontine nuclei, which likely play an important role in locomotion control, they developed dopamine-resistant gait and balance disorders [88]. Thus, future studies could also consider using the NHP with subthreshold dopaminergic lesions to evaluate the role of other neurotransmitters by creating changes in a second system using lesions (pharmacological or toxic) or activation (optogenetics, DBS). Amenable systems to investigate might include cholinergic (basal nucleus, striatum, PPN), noradrenergic (locus coeruleus, sub-coeruleus), glutamatergic (PPN, thalamus, STN) and others, potentially combined with surface EEG or multiple DBS lead recordings in differing sites that could be utilised in complex modeling.

### Interventional and nested clinical trials

There have been only a few symptomatic trials targeting FOG in PD, which have utilised a range of interventions including exercise, cognitive training, DBS, DBS with methylphenidate, noradrenergic reuptake inhibitor (atomoxetine), istradefylline, and amantadine (for review see Gao et al. 2020 [89] and Delgado-Alvarado et al. 2020 [90]). These studies have reported varying levels of success but clearly may have been flawed by the issues regarding assessment and measurement covered above. It is very much hoped that moving forwards, we will see further studies evaluating symptomatic therapies and even some that might aim to delay or prevent the onset of FOG potentially through the targeting of key predictors such as anxiety or through the early implementation of physical/cognitive training programs in enriched cohorts of ‘at-risk’ non-freezers [91, 92]. In addition to standardising the assessments and measurement of FOG, it would be potentially very useful to promote the concept of nesting planned trials in larger prospective meta-analyses. It is unlikely that any single FOG trial or research study will have a significant number of participants, but the field could agree to utilise the data by collecting individual participant data into pre-defined prospective meta-analyses, in which data from these studies can be included before the results are known. This approach has been very successful in peri-natal medicine and represents a more efficient use of any data generated [93]. Indeed, well planned prospective analyses would reduce problems with any retrospective aggregate analysis, such as reporting bias or discordant data definitions [94]. Such a study design would also be relatively inexpensive compared to creating a larger dedicated prospective trial and may offer the potential to get patients to add their own data, supported by an online evaluation resource (see above), if such a platform existed.

## Future directions

The role of this paper is to provide inspiration to better understand FOG by highlighting the opportunities that exist whereby standardised data could be integrated within novel computational modeling approaches to test hypotheses. It must be emphasised that there are many barriers to such an integrated approach, including the feasibility of coordinating standardised activity across multiple international centres; creating shareable databases; establishing effective collaborations across disciplines that have limited experience in working together; and of course, how such studies would be funded. However, if successful, it is envisioned that computational models incorporating neuroanatomical, neurotransmitter, neurophysiological and clinical domains could be used to generate potential pathophysiological pathways to explain where normal gait breaks down leading to discrete freezing episodes, including the influence of triggers (e.g., turning, cognitive load). Understanding the neurobiology underpinning such events could then inform targeted, hypothesis-driven therapies, such as modulating neurotransmitter levels or changing neural firing patterns on demand with DBS. Similarly, such computational models could be used to predict the onset of freezing in previous non-freezers where rather than evaluating patients in a simple conversion study, multi-level datasets would be able to detect the inflexion point for critical parameters (e.g., kinematics, cognitive performance, neuroimaging or neurophysiological changes) that would indicate the progression of underlying pathogenic mechanisms as they became involved in the development of FOG. Such insights would allow refinements in the therapeutic pipeline and potentially personalised medicine approaches [95]. Whilst seeming like an over-reach, many of the proposals outlined here are achievable but would require clear leadership and a global consensus, potentially coordinated by professional organisations such as the International Parkinson and Movement Disorder Society or agencies such as the National Institutes of Health working with members of the International Freezing of Gait Workshop. Indeed, without such a paradigm shift, it is possible that major advances in the field will not be realised and that we will remain ‘out of step’ with the needs of our patients.

## Conclusions

Advancing our understanding and treatment of FOG will require a change in our approach, including:

- Standardised definitions and measurements
- Multi-modal data collection integrating techniques such as imaging, neurophysiology and clinical biomarkers
- Cooperation between clinicians and data scientists.

## Data Availability

Not applicable.

## Abbreviations

FOG: Freezing of gait
MRI: Magnetic resonance imaging
PET: Positron emission tomography
EEG: Electroencephalography
EMG: Electromyography
DBS: Deep brain stimulation
NHP: Non-human primate
VR: Virtual reality
